# Effects of a Co-Design–Based Invitation Strategy on Participation in a Preventive Health Check Program: Randomized Controlled Trial

**DOI:** 10.2196/25617

**Published:** 2021-03-10

**Authors:** Trine Thilsing, Lars Bruun Larsen, Anders Larrabee Sonderlund, Signe Skaarup Andreassen, Jeanette Reffstrup Christensen, Nanna Herning Svensson, Marie Dahl, Jens Sondergaard

**Affiliations:** 1 Research Unit of General Practice Department of Public Health University of Southern Denmark Odense C Denmark; 2 Steno Diabetes Center Zealand Holbaek Denmark; 3 Steno Diabetes Center Odense Odense Denmark; 4 Research unit of User Perspectives and Community-based Interventions Department of Public Health University of Southern Denmark Odense C Denmark; 5 Vascular Research Unit Department of Surgery Regional Hospital Central Denmark Viborg Denmark; 6 Department of Clinical Medicine Aarhus University Aarhus Denmark

**Keywords:** participation rate, prenotification, invitation letter, co-design, preventive health checks, primary care

## Abstract

**Background:**

Preventive primary care programs that aim to reduce morbidity and mortality from lifestyle-related diseases are often affected by low-to-moderate participation rates. Improving participation rates is essential for clinical effectiveness and cost-effectiveness. In 2016-2017, we conducted a pilot study (TOF pilot1) for a preventive primary care intervention (TOF is the Danish abbreviation for “Early Detection and Prevention”). Among 8814 invited patients, 3545 (40.22%) consented to participate, with the highest participation rates among women and patients with higher income, education, and employment.

**Objective:**

The aim of this study was to evaluate the effects of a revised invitation strategy on invitation comprehensibility, the overall participation rate, and participant demography. The new strategy specifically targeted men and patients of low educational attainment.

**Methods:**

This study was embedded in the second TOF pilot study (TOF pilot2, initiated in October 2018) that tested an adjusted intervention. The revised invitation strategy comprised a prenotification postcard and a new invitation that specifically targeted men and patients of low educational attainment. The new invitation was developed in a co-design process that included communication professionals and target-group representatives. The study sample consisted of 4633 patients aged between 29 and 59 years, who resided in one of two municipalities in the Region of Southern Denmark. Eligible patients were randomly assigned to one of four invitation groups. The control group (Group 1) received the original invitation used in TOF pilot1. The intervention groups received the original invitation and the prenotification postcard (Group 2), the new revised invitation and the prenotification postcard (Group 3), or the new invitation but no prenotification postcard (Group 4).

**Results:**

Overall, 2171 (46.86%) patients consented to participate. Compared to the control group, participation rates increased significantly in all three intervention groups (all *P*<.001). Participation across the three intervention groups increased for women and men, as well as for patients with high and low educational attainment and high and low family income. The largest relative increase in participation rates occurred among men, patients with low educational attainment, and patients with low family income. No increase in participation was detected for unemployed patients or patients of non-Danish origin. Most participants found the original (813/987, 82.37%) and new (965/1133, 85.17%) invitations easy to understand with no significant difference (*P*=.08) in comprehensibility between invitations.

**Conclusions:**

The results suggest that participation in preventive primary care interventions can be greatly increased by implementing a co-design–based invitation strategy that includes prenotification postcards and targeted invitations. Although firm conclusions cannot be made from this study, the observed increased participation rates for men and patients of low socioeconomic status may be relevant in programs that aim to reduce social inequality in health.

**Trial Registration:**

ClinicalTrials.gov NCT03913585; https://clinicaltrials.gov/ct2/show/NCT03913585

## Introduction

### Background

Preventive primary care health checks that aim to reduce lifestyle-related morbidity and mortality often have only moderate-to-low participation rates (<50%) [1-4]. In addition, the rate of uptake appears to be unevenly spread across the population. Specifically, patients who are most likely to participate in preventive health checks are those who have a higher socioeconomic status, are older, are female, or have a lower than average prevalence of cardiovascular risk factors [3,5,6].

Important factors that facilitate participation include the mode of invitation, patient awareness of the given preventive program, and clarity of the program’s purpose [7-9]. For example, a written invitation is often the first point of contact between program providers and the patient, thus representing a vital element in recruitment. Previous studies have assessed the effects of invitation content and mode of distribution on participation rate. A US study targeting Hispanic employees found that participation in a worksite dietary intervention increased when the initial advertisement (a flyer) was supplemented with a personalized letter to individual workers. Tailoring the letter further by including heart disease risk statistics for Hispanics rather than the general population, however, had no additional effect on participation [10]. By contrast, Sallis et al increased participation in a health check intervention from 29.3% to 33.5% by revising the invitation with insights from behavioral science, including simplification, action-focused behavioral instructions, and personal salience, and specifically addressing implementation intentions [11]. Other tactics, including emphasizing support from a funding agency, incorporating endorsements from senior-position health professionals as opposed to junior-position health professionals [12], and tailoring the invitation with information about patient cardiovascular disease risk, had no effect [13]. Finally, in a recent study by Koitsalu et al, higher participation rates in a cancer screening program were associated with the use of prenotification postcards and reminders. The study also assessed invitation length but found no effect on participation [8]. While these studies report on a relatively broad range of specific invitation methods that seem to vary somewhat in effectiveness, the evidence ultimately indicates the potential for boosting intervention participation by focusing on both the overall invitation strategy and individual invitation components.

### Specific Basis for Our Study

In a recent study (TOF pilot1), we tested the feasibility and acceptability of a step-wise approach to preventive primary care health checks [14]. TOF is the Danish abbreviation for “Early Detection and Prevention.” The TOF intervention centers on a two-step process as follows: (1) the identification and stratification of the at-risk population through a participant risk-assessment questionnaire combined with health information from electronic patient records at general practitioners (GPs); (2) an offer of targeted and cohesive preventive services to the high-risk population.

In the TOF pilot1 study, a random sample of 8814 patients (aged 29-59 years) from 47 GP clinics was invited to take part in the study [2,14,15]. An invitation to participate was sent to each patient’s digital mailbox, followed by two reminders 2 weeks apart in the event of nonresponse. The digital mailbox is an online system provided by the Danish government for secure communication between individuals and public authorities/other trusted organizations. Almost all (>92%) Danish citizens aged 15 years or above have a digital mailbox [16].

A total of 3545 (40.22%) invited patients consented to take part in the study. Consistent with other similar studies, women and patients with higher income, education, and employment rates were most likely to participate [2].

In an attempt to increase general participation rates as well as participation specifically among underrepresented patient groups at possible increased risk of lifestyle-related diseases (males and patients with low educational attainment) [17,18], we revised the invitation strategy, taking a pragmatic co-design approach. To this end, we engaged communication professionals as well as target-group representatives [19]. This paper reports on the effects of the revised invitation strategy on invitation comprehensibility, the overall participation rate, and participant demography.

## Methods

### Context of the Study

This study is nested within the second TOF pilot study (TOF pilot2) that tests the feasibility and acceptability of an adjusted version of the TOF intervention. The study has been registered on ClinicalTrials.gov (NCT03913585).

### Adjustments to the Invitation Strategy

Prior to study commencement, the TOF invitation strategy was revised by (1) adding a prenotification postcard sent to prospective participants 2 weeks prior to the digital invitation, and (2) designing a new invitation based on the results of a co-design approach [19]. The prenotification postcard and the original and new TOF invitations (all translated into English) are presented in Multimedia Appendix 1, Multimedia Appendix 2, and Multimedia Appendix 3.

### Prenotification Postcard

The purpose of the prenotification postcard was to create awareness about the upcoming invitation and the TOF intervention in general. It urged recipients to keep an eye on their digital mailbox during the next couple of weeks as “something important is on its way.” The prenotification also contained brief information about the full intervention, including the possibility of receiving a preventive health check, and was signed by the patients’ GP, the municipality, and the Region of Southern Denmark.

### Invitation

The original and new invitations were designed as one-page PDF files written in Danish. Both included the contact details of the project coordinator and a hyperlink to the study webpage where additional information about the study could be accessed, including a short animated film outlining the individual steps of the intervention. Both invitation versions also included a link to a web-based digital support system through which participants could provide their informed consent to participate. The invitations were addressed to individual patients and were signed by the patients’ GP, the municipality, and the Region of Southern Denmark.

### Development of the New Invitation by a Co-Design Approach

The new invitation was developed in a pragmatic co-design process in collaboration with communication professionals, purposely sampled representatives from the target population, and the Men’s Health Society [20], a multidisciplinary organization dedicated to the field of men’s health in all its aspects.

First, three revamped versions of the invitation were developed by communication professionals and on the basis of current knowledge on facilitators and barriers for participation in preventive health checks [9,11]. All versions were designed to include clear and unambiguous information about the intervention, action-focused behavioral instructions (pictogram), and information on target-group selection (scarcity). A pictogram was included to clearly depict the individual steps of the intervention (when, where, and how), with the aim to rehearse the cognitive link between behavior and context [21]. A special effort was made to keep sentences short, concise, and free of jargon [22]. The exact wording of the invitation was inspired by recent Danish preventive programs focusing on men and socially deprived groups [23,24], and by specific recommendations from the Men’s Health Society.

Thereafter, the three new invitations were discussed and tested for content and comprehensibility in a focus group. Focus group members were purposely sampled to include men and people with low educational level. The recruitment process comprised advertisements on Facebook and Instagram, which were targeted at people aged 29 to 59 years with low educational attainment. In addition, attendees at “Meeting Place for Men” in the city of Sønderborg [25] (a social connectedness initiative for men) were contacted directly. The resulting focus group comprised 10 people between 34 and 57 years old (three women and seven men). Eight focus group members had no formal education beyond secondary school, one was a primary school teacher, and one was a printmaker. None of the focus group members were eligible to participate in the TOF pilot2 study as they resided outside the participating municipalities.

A semistructured interview guide was developed by a multidisciplinary research team and communication professionals. The guide included thematic open- and closed-ended questions on facilitators and barriers for receiving information through the digital mailbox, on general perceptions of health, and on the specific content and comprehensibility of the three invitations [26]. Before the meeting, all focus group members were asked to study the three invitations. During the meeting, all participants were encouraged to provide input.

The focus group meeting was filmed and transcribed. Strong action-oriented comments, points, and statements about the content, wording, and design of the three invitations were identified and used in the development of the final invitation.

### Study Design

The effect of the revised invitation strategy on overall participation and participant demography was tested in a randomized controlled trial nested within the TOF pilot2 study. Initially, a total of 61 GPs from 22 GP clinics in two municipalities in the Region of Southern Denmark (Haderslev and Middelfart) were invited to take part in the study. Subsequently, the target group was selected from the patient registries of the participating GP clinics and comprised patients born between 1959 and 1988 (aged 29-59 years). The chosen age range resembles age ranges used in previous lifestyle interventions in primary care [27-30] and was determined on the rationale that people in this age group may achieve the greatest health benefits from improvements in lifestyle. Patients were excluded if they lived outside the municipalities of Haderslev or Middelfart, if they did not have a digital mailbox (<5% of the target population) [16], or if their name and address were unlisted. Patients from the municipality of Haderslev who were invited to the first TOF pilot study (TOF pilot1) were also excluded.

Before study commencement, all eligible patients were randomly assigned to one of four invitation groups and thus received one of the following: (1) the original invitation used in the first TOF pilot study (control group, Group 1), (2) a prenotification postcard and the original invitation (Group 2), (3) a prenotification postcard and the new invitation (Group 3), and (4) the new invitation but no prenotification postcard (Group 4).

In order to avoid cross-contamination, patients living together were randomized to the same invitation group. Randomization was done using Stata (refer to the subsection Sample Size Calculation, Randomization, and Statistical Analysis).

On October 8, 2018, the prenotification postcard was sent by standard mail to patients in Groups 2 and 3. Two weeks later on October 22, 2018, invitations were sent to the digital mailboxes of all eligible patients (Groups 1-4). In the event of nonresponse, the invitation was followed up with two digital reminders sent 2 weeks apart. The reminders were identical to the first invitation, except for a brief sentence that informed the participant that this was a reminder. The deadline for providing informed consent to participate was December 3, 2018 (6 weeks after the invitation was first sent out).

Immediately following consent, the participant was redirected from the digital support system to an electronic questionnaire that included the following question on invitation comprehensibility: “The information about the project included in the digital invitation was.” The answer options were as follows: “Easy to understand,” “Fairly understandable,” and “Difficult to understand.”

### Dependent Variable

Patients were defined as participants or nonparticipants based on whether they had provided informed consent to participate in the study.

### Independent Variables

Invitation group (Groups 1-4), invitation type (original/new), and prenotification postcard (yes/no) were defined as described above. Participants’ evaluation of invitation comprehensibility was dichotomized as 1 (“easy to understand”) or 0 (“fairly understandable/difficult to understand”).

### Register-Based Data

Information on sex, age, country of origin, educational level, employment status, and family income was retrieved from the national Danish Bureau of Statistics (Statistics Denmark) and linked with individual patients’ Danish Personal Identification number (CPR).

Participant age was determined at the time of invitation and categorized in 10-year age brackets. Country of origin was retrieved for the year 2018 and categorized as Danish, Western, or non-Western. Western countries included countries in the European Union and associated countries, as well as the United States, Canada, Australia, and New Zealand. Non-Western countries included the European countries of Albania, Bosnia and Herzegovina, Belarus, Yugoslavia, Kosovo, Macedonia, Moldova, Montenegro, Russia, Serbia, Soviet Union, Turkey, and Ukraine; all countries in Africa, South and Central America, and Asia; and all countries in Oceania (except Australia and New Zealand). Stateless persons were also defined as non-Western. The highest attained educational level was retrieved for October 2018 and categorized as secondary school, high school, vocational education, higher education, or higher education-master’s level. Subsequently, the highest educational attainment was dichotomized (low educational attainment [yes/no]: yes = secondary school; no = high school, vocational education, higher education, or higher education-master’s level). Employment status was retrieved for November 2018 and categorized as employed, self-employed, unemployed/on benefits, social welfare recipient, or other. In Denmark, all unemployed workers are eligible to receive social welfare benefits, whereas unemployment benefits are accessible only to citizens who have been unemployed for less than 2 years and who are members of a voluntary unemployment benefit fund. The final group (“others”) represents, for example, unemployed persons from a family that relies on one income only. For all analyses, employment status was dichotomized (unemployed [yes/no]: yes = unemployed/on benefits, social welfare recipients, or other; no = employed or self-employed). Family income was retrieved for 2013-2018, defined by the mean annual net income of the household, and was categorized in quartiles. Subsequently, family income was dichotomized (low income [yes/no]: yes = lowest quartile; no = above the lowest quartile).

### Sample Size Calculation, Randomization, and Statistical Analysis

Based on results from a previous study that employed a similar approach of prenotification postcard followed by invitation [8], we estimated that the intervention could achieve a 6% increase in the participation rate (ie, 46% compared with 40% in the control group) [2]. The study was therefore designed to detect a 6% difference in the participation rate between control and intervention groups with 80% power and 5% significance. This required a total sample size of 4404 (1101 per group) patients.

Randomization was performed by participant address, assigning random numbers to the cohort using the generate rannum = uniform() command in Stata. Subsequent allocation to create approximately equally sized groups was performed using the egen recruitmentgroup = cut(rannum), group(4) command.

Descriptive statistics have been used to present the study sample. Generalized linear models (binreg) were run to assess the effects of invitation mode and individual invitation elements on participation rates. Adjusted analyses accounted for age and sex.

Chi-square analyses were performed to compare participation rates in each of the three intervention groups to that in the control group overall and in sociodemographic subgroups. Generalized linear models (binreg) were run to calculate risk ratios (RRs) and 95% CIs. RR was chosen over odds ratio (OR) as OR tends to exaggerate the estimate of the relationship between an exposure and an outcome in cases where there is an association [31].

Generalized linear models (binreg) also assessed the association between invitation type (original or new) and level of comprehensibility (“easy to understand” or “fairly understandable/difficult to understand”). The significance level was set at *P*<.05.

All statistical analyses were performed on secure servers at Statistics Denmark using Stata version 16.0 (Statacorp).

### Ethics Approval and Consent to Participate

The study was approved by the Research & Innovation Organisation, University of Southern Denmark (18/32742), and the TOF pilot2 study was registered at ClinicalTrial.gov (NCT03913585) [32]. According to Danish regulations (Act on Research Ethics Review of Health Research Projects [section 14.2]), this study did not need approval from a health research ethics committee as no research on human tissue or other biological material was performed. The study complies with the Helsinki Declaration by requiring informed consent from participants.

Focus group members received compensation for transportation costs but were not remunerated for their participation in the meeting. Participants in the TOF pilot2 study did not receive any remuneration or compensation.

## Results

### Assignment to Invitation Groups and Sociodemographic Characteristics of the Invitation Groups

A total of 15 GPs from four clinics took part in the study. Of 6347 patients born between 1959 and 1988, 4633 were eligible to participate in the study. Random assignment placed 1151 patients in Group 1 (original invitation, no postcard [control]), 1156 in Group 2 (original invitation plus postcard), 1148 in Group 3 (new invitation plus postcard), and 1178 in Group 4 (new invitation, no postcard).

Table 1 shows the sociodemographic characteristics of all 4633 patients who were invited to take part in the study. Overall, the four invitation groups did not differ in any demographic characteristics.

**Table 1:** Sociodemographic characteristics and mode of invitation among patients invited to participate in a preventive primary care program for lifestyle-related diseases (TOF pilot2).

Mode of invitation		Group 1: Original invitation (control) (n=1151, 24.84%), n (%)	Group 2: Original invitation + postcard (n=1156, 24.95%), n (%)	Group 3: New invitation + postcard (n=1148, 24.78%), n (%)	Group 4: New invitation (n=1178, 25.43%), n (%)	Total (n=4633, 100%), n (%)
**10-year age groups (missing n=0)**						
	29-39 years	302 (26.24)	315 (27.25)	321 (27.96)	309 (26.23)	1247 (26.92)
	40-49 years	441 (38.31)	437 (37.80)	456 (39.72)	442 (37.52)	1776 (38.33)
	50-60 years	408 (35.45)	404 (34.95)	371 (32.32)	427 (36.25)	1610 (34.75)
**Sex (missing n=0)**						
	Male	545 (47.35)	533 (46.11)	561 (48.87)	560 (47.54)	2199 (47.54)
	Female	606 (52.65)	623 (53.89)	587 (51.13)	618 (52.46)	2434 (52.46)
**Country of origin (missing n=21, 0.45%)**						
	Denmark	1044 (91.26)	1042 (90.37)	1040 (90.99)	1064 (90.78)	4190 (90.85)
	Western	32 (2.80)	38 (3.30)	45 (3.94)	42 (3.58)	157 (3.40)
	Non-Western	68 (5.94)	73 (6.33)	58 (5.07)	66 (5.63)	265 (5.75)
**Highest educational attainment (missing n=155, 3.34%)**						
	Secondary school	196 (17.61)	189 (16.83)	183 (16.56)	190 (16.71)	758 (16.93)
	Highschool, vocational education, higher education, or higher education-master’s level	917 (82.39)	934 (83.17)	922 (83.44)	947 (83.29)	3720 (83.07)
**Employment status (missing n=15, 0.32%)**						
	Unemployed/on benefits, social welfare recipients, or other^a^	208 (18.15)	207 (17.94)	213 (18.60)	215 (18.33)	843 (18.25)
	Employed or self-employed	938 (81.85)	947 (82.06)	932 (81.40)	958 (81.67)	3775 (81.75)
**Family income (missing n=21, 0.45%)**						
	Lowest quartile	260 (22.73)	276 (23.94)	270 (23.62)	288 (24.57)	1094 (23.72)
	Greater than the lowest quartile	884 (77.27)	877 (76.06)	873 (76.38)	884 (75.43)	3518 (76.28)

^a^“Other” represents, for example, unemployed persons from a family that relies on one income only.

### Participation

**Figure 1:**
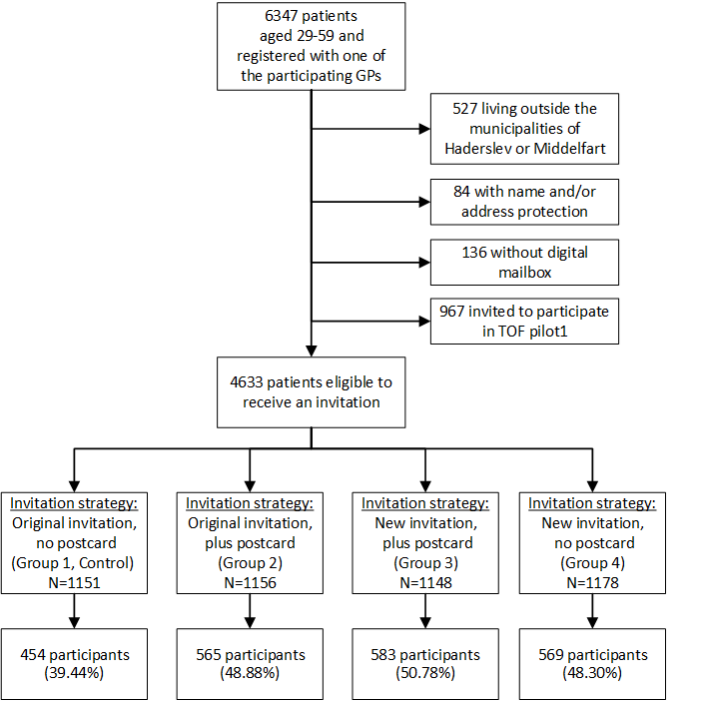
Flow diagram of a preventive primary care program (TOF pilot2) from initial sampling of patients to participation rates in each of four invitation groups. GP: general practitioner.

A total of 2171 (46.86%) out of 4633 invited patients consented to participate in the study. Participation rates ranged from 39.44% (454/1151) to 50.78% (583/1148) across the four invitation groups. The participation rate for patients who received the original invitation alone (control group) (39.44%) was comparable to the participation rate obtained in TOF pilot1 (40.22%) [2]. Figure 1 shows the flow of patients from sampling to participation in the TOF pilot2 study.

Compared to participants in Group 1, who received the original invitation alone, participation rates were higher for Group 2 (original invitation plus prenotification postcard), Group 3 (new invitation plus prenotification postcard), and Group 4 (new invitation alone). These differences in participation rates remained highly significant after adjustments for age and sex (Table 2). The highest rate of participation was achieved in Group 3. Differences in participation rates between the three intervention groups (Groups 2, 3, and 4), however, did not reach statistical significance (Group 2 vs 3: *P*=.36; Group 2 vs 4: *P*=.78; Group 3 vs 4: *P*=.23).

**Table 2:** Analysis of associations between participation, mode of invitation, and invitation elements among patients invited to take part in a preventive primary care program for lifestyle-related diseases (TOF pilot2).

Variable			Sample size (n)	Model 1 (crude)		Model 2 (adjusted for age and sex)	
				RR^a^ (95% CI)	*P* value	RR (95% CI)	*P* value
**Mode of invitation**							
	Original invitation (control)		1151	1 (0)^b^	N/A^c^	1 (0)^b^	N/A
	Original invitation + postcard		1156	1.24 (1.13-1.36)	<.001	1.23 (1.12-1.34)	<.001
	New invitation + postcard		1148	1.29 (1.17-1.41)	<.001	1.29 (1.18-1.41)	<.001
	New invitation		1178	1.22 (1.12-1.34)	<.001	1.22 (1.11-1.33)	<.001
**Individual invitation elements**							
	**Prenotification postcard**						
		No	2329	1 (0)^b^	N/A	1 (0)^b^	N/A
		Yes	2304	1.13 (1.07-1.21)	<.001	1.13 (1.07-1.20)	<.001
	**Invitation**						
		Original	2307	1 (0)^b^	N/A	1 (0)^b^	N/A
		New	2326	1.12 (1.05-1.19)	<.001	1.12 (1.06-1.19)	<.001

^a^RR: risk ratio.

^b^Reference group.

^c^N/A: not applicable.

Table 3 shows the difference in participation rates between the intervention groups and the control group by sociodemographic subgroups.

The three intervention groups recorded higher participation rates than the control group for both female and male patients and across all age groups. In addition, patients of any educational level, who were of Danish origin, employed, or with a family income above the lowest quartile, were more likely to participate if they received one of the three new modes of invitation. Further, with or without the prenotification postcard, the new invitation increased participation rates for patients with a family income in the lowest quartile. By contrast, none of the new modes of invitation affected participation rates for unemployed patients or patients of non-Danish (Western or non-Western) origin.

**Table 3:** Participation in a preventive primary care program (TOF pilot2) and risk ratios of participation obtained from comparing each of the new modes of invitation (Groups 2-4) to the original one (Group 1, control) overall and by sociodemographic subgroups.

Participation overall and by sociodemographic subgroups		Group 1: Original invitation (control)	Group 2: Original invitation + postcard			Group 3: New invitation + postcard			Group 4: New invitation			Total
		Value, n (%)	Value, n (%)	*P* value^a^	RR^b^ (95% Cl)	Value, n (%)	*P* value	RR (95% Cl)	Value, n (%)	*P* value	RR (95% Cl)	Value, n (%)
Overall		454 (39.44)	565 (48.88)	<.001	1.24 (1.13-1.36)	583 (50.78)	<.001	1.29 (1.17-1.41)	569 (48.30)	<.001	1.22 (1.12-1.34)	2171 (46.86)
**10-year age groups**												
	29-39 years	75 (24.83)	115 (36.51)	.002	1.47 (1.15-1.88)	134 (41.74)	<.001	1.68 (1.33-2.13)	102 (33.01)	.03	1.33 (1.03-1.71)	426 (34.16)
	40-49 years	185 (41.95)	218 (49.89)	.02	1.19 (1.03-1.37)	230 (50.44)	.01	1.20 (1.04-1.39)	217 (49.10)	.03	1.17 (1.01-1.35)	850 (47.86)
	50-60 years	194 (47.55)	232 (57.43)	.005	1.21 (1.06-1.38)	219 (59.03)	.001	1.24 (1.09-1.42)	250 (58.55)	.001	1.23 (1.08-1.40)	895 (55.59)
**Sex**												
	Male	179 (32.84)	234 (43.90)	<.001	1.34 (1.15-1.56)	248 (44.21)	<.001	1.35 (1.16-1.57)	242 (43.21)	<.001	1.32 (1.13-1.53)	903 (41.06)
	Female	275 (45.38)	331 (53.13)	.007	1.17 (1.04-1.31)	335 (57.07)	<.001	1.26 (1.12-1.41)	327 (52.91)	.008	1.17 (1.04-1.31)	1268 (52.10)
**Country of origin**												
	Denmark	432 (41.38)	530 (50.86)	<.001	1.23 (1.12-1.35)	553 (53.17)	<.001	1.29 (1.17-1.41)	541 (50.85)	<.001	1.23 (1.12-1.35)	2056 (49.07)
	Western	8 (25.00)	18 (47.37)	.054	1.89 (0.95-3.77)	16 (35.56)	.32	1.42 (0.69-2.81)	14 (33.33)	.44	1.33 (0.64-2.79)	56 (35.67)
	Non-Western	11 (16.18)	16 (21.92)	.39	1.35 (0.68-2.71)	13 (22.41)	.37	1.39 (0.67-2.85)	13 (19.70)	.59	1.22 (0.59-2.52)	53 (20.00)
**Highest educational attainment**												
	Secondary school	49 (25.00)	71 (37.57)	.008	1.50 (1.11-2.04)	80 (43.72)	<.001	1.75 (1.30-2.34)	74 (38.95)	.003	1.56 (1.15-2.10)	274 (36.15)
	Highschool, vocational education, higher education, or higher education-master’s level	393 (42.86)	484 (51.82)	<.001	1.21 (1.10-1.33)	487 (52.82)	<.001	1.23 (1.12-1.36)	484 (51.11)	<.001	1.19 (1.08-1.31)	1848 (49.68)
**Employment status**												
	Unemployed/on benefits, social welfare recipients, or other	76 (36.54)	77 (37.20)	.89	1.02 (0.79-1.31)	78 (36.62)	.99	1.00 (0.78-1.29)	77 (35.81)	.88	0.98 (0.76-1.26)	308 (36.54)
	Employed or self-employed	377 (40.19)	488 (51.53)	<.001	1.28 (1.16-1.42)	504 (54.08)	<.001	1.35 (1.22-1.48)	492 (51.36)	<.001	1.28 (1.16-1.41)	1861 (49.30)
**Family income**												
	Lowest quartile	65 (25.00)	90 (32.61)	.052	1.30 (1.00-1.71)	97 (35.93)	.006	1.44 (1.10-1.87)	100 (34.72)	.01	1.39 (1.07-1.81)	352 (32.18)
	Greater than the lowest quartile	386 (43.67)	474 (54.05)	<.001	1.24 (1.12-1.36)	485 (55.56)	<.001	1.27 (1.16-1.40)	468 (52.94)	<.001	1.21 (1.10-1.34)	1813 (51.53)

^a^*P* values for differences in the participation rate between the individual intervention groups (Groups 2-4) and the control group (Group 1).

^b^RR: risk ratio.

### Invitation Comprehensibility

A total of 2120 (97.65%) participants answered the question on invitation comprehensibility. Of these, 987 had received the original invitation and 1133 had received the new one. The response rates for these two groups were thus 96.86% (987/1019) and 98.35% (1133/1152), respectively.

Among participants who received the new invitation, 965 (85.17%) found it easy to understand. This level of invitation comprehensibility was comparable to that of the original invitation group (n=813, 82.37%, RR 1.03; 95% CI 1.00-1.07; *P*=.08). Including missing data in the “fairly understandable/difficult to understand” group did not alter these results.

## Discussion

### Focus of the Study

We investigated whether different iterations of invitation strategies might increase the participation rate in a preventive primary care intervention targeting lifestyle-related diseases. We focused on participation in general as well as participation specifically among men and people with low educational attainment.

### Effect on Participation

Each of the experimental invitation strategies greatly increased participation rates compared with the control. There were, however, no statistical differences in impact between the three intervention groups (Groups 2-4). Overall, participation rates increased for both women and men, as well as for patients of high and low socioeconomic status. However, the consistently higher RRs for men compared to women, patients with low compared to high educational attainment, and patients with low compared to high family income may indicate a larger relative effect in these groups. Firm conclusions for this effect cannot be made based on the reported results though. Taken together, the impact of an invitation strategy on participation in preventive programs like the one tested here may also have implications for other settings, such as worksite health promotion programs, which often have low/moderate participation rates [33].

The apparent effect on participation among non-Danish patients was not significant. This may be due to the rather small sample sizes, but cultural characteristics and the fact that invitations and postcards were in Danish most likely also contributed [34,35]. Similarly, the relatively low participation rates for unemployed patients may signify greater participation barriers in this group. For example, unemployment has previously been shown to be associated with poorer mental and physical health, which may impede the likelihood of taking part in research studies [36,37]. We also note, however, that the revised invitation was designed to target men and patients of low educational attainment. As such, the purposely sampled focus group for invitation design did not include unemployed patients or patients of non-Danish origin specifically. Involving patients from these target groups in future design processes might allow for invitation strategy adjustments to accommodate these groups as well.

### Specific Invitation Elements

Supplementing both the original and revised invitations with the prenotification postcard increased participation rates. Of particular note, the notoriously hard-to-reach youngest age group (29-39 years old) also responded well to this mode of invitation. These results are consistent with previous research showing higher participation and odds of response to questionnaires when the study invitation is preceded by a prenotification [8,38]. The specific content of the postcard likely contributed further as the teaser sentence “Something important is on its way – remember to keep an eye on your digital mailbox” may have primed participants to be more attentive to their digital mailbox and the upcoming invitation. Indeed, a systematic review on methods to increase responses to postal and electronic questionnaires revealed that using teasers on the envelope can increase participation [38].

The new invitation comprised action-focused behavioral instructions (pictograms), shorter sentences, and jargon‐free language. Formatting invitation content in this way has been demonstrated to facilitate research participation in both the general population as well as hard-to-reach subgroups, including people with intellectual disabilities [22]. In other words, the deliberate focus on creating an easy-to-grasp lay-person invitation may have contributed to engaging a broader audience.

Compared to the original invitation, the heading of the new invitation included a clear incentive (“Get a free health check”) coupled with an action-focused instruction (“Use five minutes on a questionnaire”). Although the effect of such specific wording is difficult to assess, past research would suggest that it may have influenced participation. For instance, Sallis et al increased participation in a National Health Service health check program by using behavioral instructions and concrete statements in the invitation [11].

In this study, most participants found both invitations easy to understand with no significant difference in comprehension between the two. As invitation comprehensibility was only assessed in patients consenting to participate, it cannot be ruled out that nonparticipation may be related to invitation comprehensibility, that is, people might have declined the invitation because it was not sufficiently clear to them. Nonetheless, results from a study on female nonparticipants in a screening program for cardiovascular diseases and diabetes revealed that although the participants believed they understood the screening invitation, they seemed unaware of what the examination entailed [39]. Thus, self-reported understanding of similar written information may be somewhat biased.

While we also assessed other potential factors for nonparticipation besides invitation comprehensibility (eg, motivation and time constraints) [9], these results will be published elsewhere as they are deemed beyond the scope of this article.

### Clinical Implications

Preventive programs rely on high uptake to optimize clinical effectiveness and cost-effectiveness [40]. However, in many studies, patient uptake is disproportionately higher for women than men and for patients of high than lower socioeconomic status [2,3,5,6]. To this end, initiatives to increase uptake among men and among men and women of low socioeconomic status should be prioritized to mitigate inequality in health.

Results from this study indicate that targeting men and patients of low educational attainment led to an increase in participation not only in these groups, but also among women and patients of high educational attainment. Despite this, co-design processes, like the one tested here, may still be relevant in efforts focusing on social inequality in health, as the relative increase in participation rates appeared to be higher for men, patients with low educational attainment, and patients with low family income.

Further, in order to reach the most socioeconomically disadvantaged groups, the invitation strategy should probably be combined with other more individual-oriented recruitment approaches [41,42]. Recent evidence suggests that the use of support workers in general practice with the specific aim to increase uptake of preventive health checks may greatly increase participation among patients from deprived areas and among minority groups [43]. In addition, Cook et al found that invitation by telephone was highly effective for recruiting patients from specific ethnic minority groups [44].

### Digital Versus Paper-Based Invitations

In this study, we used a digital mailbox and a web-based digital support system to distribute invitations and obtain informed consent. Although access to both systems required a two-phase log in, results from the TOF pilot studies showed participation rates comparable to those obtained in other studies that relied on paper-based invitations [2,4,11]. This may in part be explained by the fact that the digital mailbox is a trusted and familiar mode of communication between the individual citizen and municipal, regional, and national authorities in Denmark. Another advantage relates to cost. For example, Ebert et al found that web-based invitations were more cost-effective (by a factor of 10) than paper-based ones and that nonrespondents were demographically similar in the two groups, indicating low risk of selection bias [45]. In this study, combining the new invitation with a prenotification postcard did not outperform the new digital invitation alone in terms of participation rates. Therefore, the additional cost associated with distributing the prenotification postcard should be considered in any relevant invitation strategy.

### Strengths and Limitations

In this study, sample size calculations were based on the expected overall increase in the participation rate rather than specific participation rates in socioeconomic subgroups. Repeating the study with a larger population might reveal an increase in participation rates for patients of non-Danish origin. In addition, a larger study would reveal whether the observed relative increase in participation rates for men compared to women and for patients with low compared to high educational attainment and family income is replicable.

From the results, it was not possible to assess which specific aspects of the revised invitation and/or prenotification postcard drove the observed effects. Using a more rigorous and theory-based co-design procedure may lead to more insights into these aspects. In addition, it would be relevant to test the potential effect of combining different recruitment strategies in future research.

As nearly all (98%) Danish citizens are registered with a GP [46], the study sample resembled the general Danish population for this particular age group. However, since patient sampling was based on GP clusters, differences in participation rates between these clusters cannot be excluded, and future studies may consider looking into this.

We randomized our sample by household to ensure that patients living together were invited in the same way. This procedure along with sample representability provided the study with high internal as well as external validity. In addition, this study provides important new insights into the potential effects of making a special effort when it comes to invitation strategies for preventive health checks. Further, tailoring the invitations to specific groups by employing co-design procedures may help attenuate inequalities in health.

### Conclusion

The results of this study showed that high improvements in participation rates in a preventive health check intervention may be obtained by taking a co-design approach to the invitation strategy that involves communication professionals and target-group representatives. In particular, the increased participation of men and patients of low socioeconomic status indicates the potential value of such initiatives to mitigate inequalities in health.

## Appendix

Multimedia Appendix 1 Original invitation.

Multimedia Appendix 2 New invitation.

Multimedia Appendix 3 Prenotification postcard.

Multimedia Appendix 4 CONSORT 2010 checklist.

## Abbreviations

GP: general practitioner
OR: odds ratio
RR: risk ratio
TOF: Early Detection and Protection Project (translated Danish abbreviation)
