# Supplementary material for: Effects of a Co-Design–Based Invitation Strategy on Participation in a Preventive Health Check Program: Randomized Controlled Trial
Source: JMIR Public Health Surveill. 2021 Mar 10;7(3):e25617. doi: 10.2196/25617 (PMC7991992; doi:10.2196/25617)
Supplement: Multimedia Appendix 1 [file publichealth_v7i3e25617_app1.pdf]

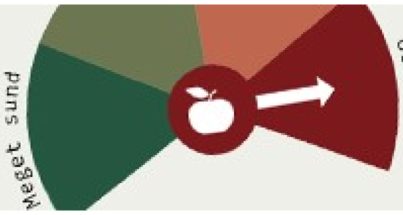


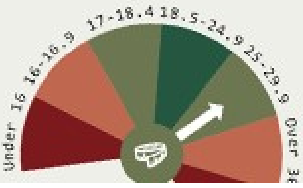


Name Surname
Address
Postal code, City

Date

# Get a personal health profile – participate in Project Early Detection and Prevention

A personal health profile provides insight into your current health condition as well as any specific areas where you might be able to improve your lifestyle. If needed, you will also be offered a health check up at your GP or at your municipal health center. To get a personal health profile, you need to sign up to participate in this project, and at a later stage, fill you a questionnaire about your lifestyle.

## Why have you received this letter?

You have been selected to participate because you live in Varde Municipality and were born between 1959 and 1988. The goal of the present project is to prevent lifestyle-related diseases such as COPD, diabetes, and cardiovascular disease. The project ends in July 2019.

## This is how you sign up to participate in the project:

1. Click the button below
2. Log on with your NemID
3. Give your consent

I WANT TO SIGN UP

If that doesn't work, you can go to tof.sundhedsmappe.dk and log on with your NemID

If you choose not to participate, please let us know why by clicking here.

## Your participation is important

Project Early Detection and Prevention is a collaborative research project. You will get a personal health profile. In doing so, you will also contribute to valuable knowledge about how general practitioners and municipality can best offer targeted prevention interventions to citizens who need it.

We encourage you to participate, regardless of whether you are healthy or currently in treatment. If we do not hear from you within 7 days, we may take the liberty to contact you again.

104256599

**Learn more about the project here:**
**www.projekttof.dk or contact the project coordinator at 23 36 24 68**

***Kind regards,***
The general practice *(Name),* The Municipality *(Name)* and The Region of Southern Denmark

*All data collection, processing, and storing as it relates to Project Early Detection and Prevention, will be handled in accordance with the GDPR (article 6, stk. 1, litra a) og article 9, stk. 2, litra a) as well as health care legislation (§ 42 d, stk. 1). The project has been approved by the University of Southern Denmark (J.nr: 18/32742).*
*We apologize in advance if our invitation is in any way offensive. Please disregard this invite if that is the case. You can click the 'I want to sign up' button and then click the 'I do not consent' button. This way, you will receive no further mail from us.*
