# Supplementary material for: Effects of a Co-Design–Based Invitation Strategy on Participation in a Preventive Health Check Program: Randomized Controlled Trial
Source: JMIR Public Health Surveill. 2021 Mar 10;7(3):e25617. doi: 10.2196/25617 (PMC7991992; doi:10.2196/25617)
Supplement: Multimedia Appendix 2 [file publichealth_v7i3e25617_app2.pdf]

Name Surname
Address
Postal code, City

Date

Get a free health check - spend five minutes of your time filling out a questionnaire.

Dear Name Surname
The general practice, *Name*, are participating in a research project about lifestyle-related disease prevention. The project ends in June 2019.

As you are between 29 and 59 years old, you have been randomly selected to participate in this project. As a participant, you will receive a free health assessment - all you have to do is fill out a short questionnaire. Additionally, if your health assessment indicates that you might be at increased risk of developing a lifestyle-related disease, you will get an appointment for a physical health check-up at your general practitioner.
Should you wish to participate, this is what you need to do:

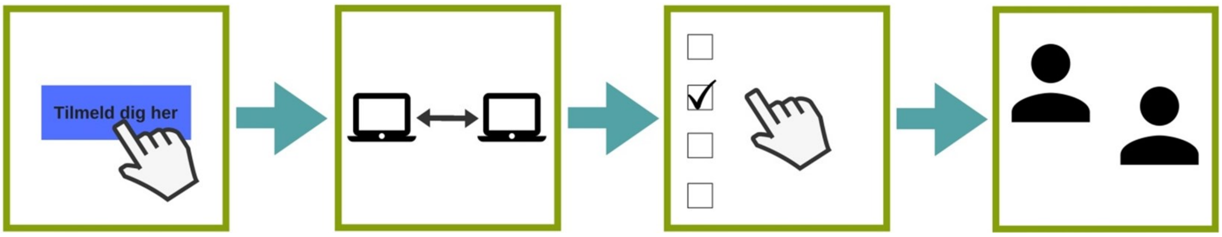

Sign up to participate and give your consent

When you have signed up and given your consent, we will collect relevant data from your patient records. This will take about 1-2 months.

When your data has been collected, we will send you a questionnaire. Once you've filled out this questionnaire, we will link your answers with your data.

When your answers and data have been linked, we will determine whether you are at increased risk of developing a lifestyle-related disease. If you are at increased risk, you will be offered a check up at your GP or your municipal health center.

Sign up

1. Click the button below.
2. Log on using your NemID
3. Give your consent.

Sign up here

To ensure data security, you need to log on using your NemID

If you do not want to participate, please click here and let us know why.
We hope you would like to participate.
We would like to encourage you to participate, regardless of whether you are currently in treatment for a condition or feel completely healthy. If you have not signed up for the project within 7 days, we may take the liberty to contact you again. The main objective of the research project is to develop an better way to identify people who are at increased risk of developing a lifestyle-related disease. Your participation will therefore contribute to an improved health care system for your neighbors, colleagues, friends, family, and fellow citizens.

Kind regards,
The general practice (*Name*), The Municipality *(Name),* The Region of Southern Denmark.

Watch a short film about the project and read more on www.projekttof.dk or call the project coordinator at 23362438.
Disclaimer
All participation in the TOF project as well as our handling of any associated data is in accordance with health care legislation and has been approved by the Data Protection Agency (Journal no. 2015570008).
We apologize in advance if our invitation is in any way offensive. Please disregard this invite if that is the case. You can click the 'Sign up here' button and then click the 'I do not consent' button. This way, you will receive no further mail from us.
