# Supplementary material for: Effects of a Co-Design–Based Invitation Strategy on Participation in a Preventive Health Check Program: Randomized Controlled Trial
Source: JMIR Public Health Surveill. 2021 Mar 10;7(3):e25617. doi: 10.2196/25617 (PMC7991992; doi:10.2196/25617)
Supplement: Multimedia Appendix 3 [file publichealth_v7i3e25617_app3.pdf]

Something IMPORTANT is on it’s way !

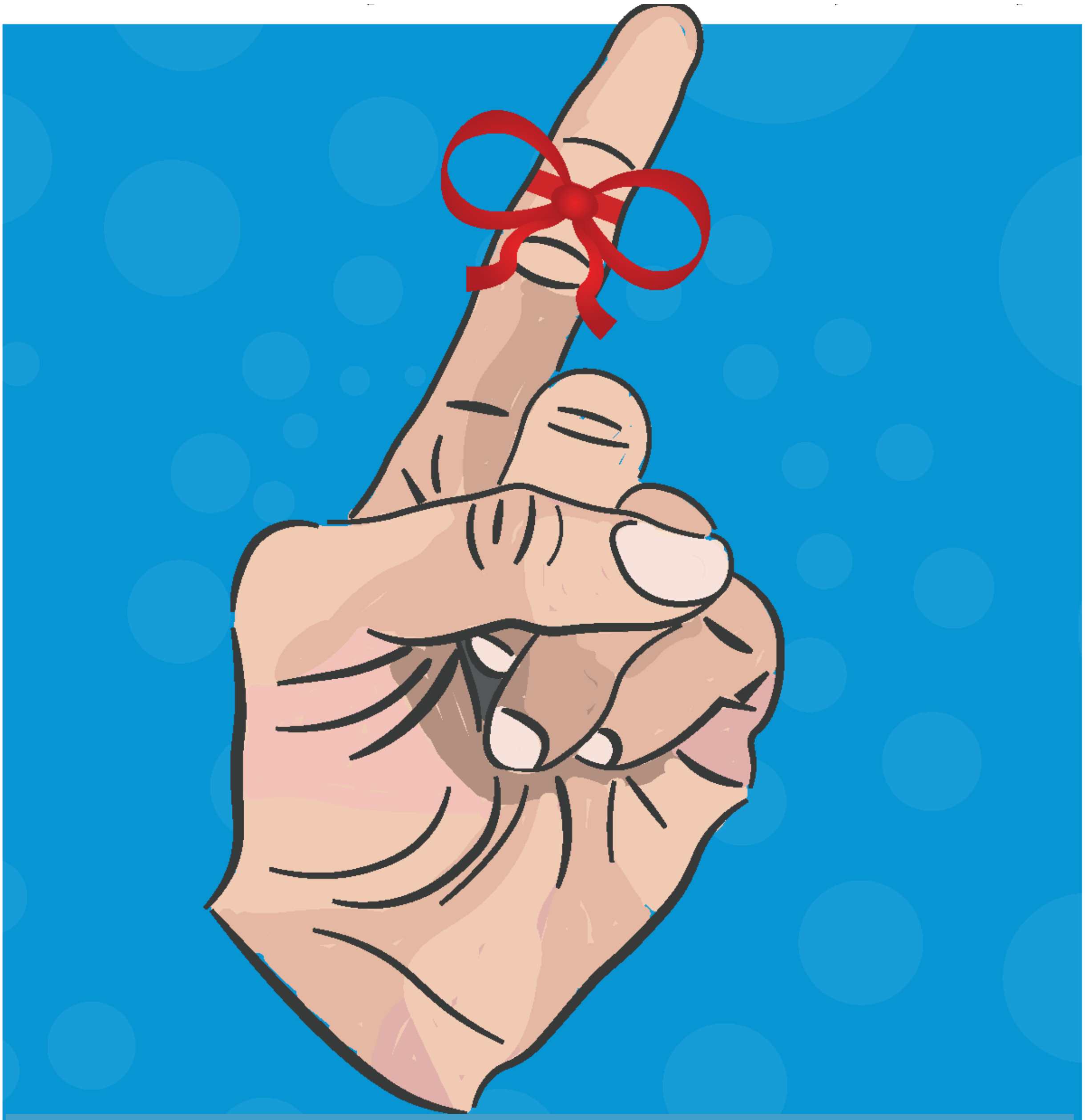

REMEMBER TO KEEP AN EYE ON YOUR DIGITAL MAILBOX

eBoks.dk

Region Syddanmark

Something IMPORTANT is on it's way in your digital mailbox

Name Surname

Address

Postal code City

Dear *Name Surname*

- You should keep an eye on your digital mailbox during the coming weeks
- You will receive an invitation to participate in a study where you can get a free health check. To participate you'll have to fill out a questionnaire. It takes approximately five minutes. By participating you will contribute with knowledge on how to efficiently prevent lifestyle-related diseases.
- We hope you are willing to participate.

Best wishes

Name of GP, Name of Municipality, University of Southern Denmark and The Region of Southern Denmark
